# Super-Pangenome by Integrating the Wild Side of a Species for Accelerated Crop Improvement

**DOI:** 10.1016/j.tplants.2019.10.012

**Published:** 2020-02

**Authors:** Aamir W. Khan, Vanika Garg, Manish Roorkiwal, Agnieszka A. Golicz, David Edwards, Rajeev K. Varshney

**Affiliations:** 1 Center of Excellence in Genomics and Systems Biology, International Crops Research Institute for the Semi-Arid Tropics (ICRISAT), Hyderabad, India; 2 School of Biological Sciences, The University of Western Australia (UWA), Crawley, WA, Australia; 3 Plant Molecular Biology and Biotechnology Laboratory, Faculty of Veterinary and Agricultural Sciences, University of Melbourne, Parkville, Melbourne, VIC, Australia

**Keywords:** pangenome, structural variations, crop wild relatives, genomics-assisted breeding, copy number variations, presence/absence variations

## Abstract

The pangenome provides genomic variations in the cultivated gene pool for a given species. However, as the crop’s gene pool comprises many species, especially wild relatives with diverse genetic stock, here we suggest using accessions from all available species of a given genus for the development of a more comprehensive and complete pangenome, which we refer to as a super-pangenome. The super-pangenome provides a complete genomic variation repertoire of a genus and offers unprecedented opportunities for crop improvement. This opinion article focuses on recent developments in crop pangenomics, the need for a super-pangenome that should include wild species, and its application for crop improvement.

## Highlights

Pangenome studies conducted so far have been limited mainly to one species and mostly cultivated accessions.
The challenge with the current pangenomes is lack of representation of genomic diversity at the genus level.
Crop wild relatives possess unearthed genetic diversity that has been lost during domestication and breeding.
Pangenomics of crop wild relatives is the way forward to catalogue the complete gene repertoire of a genus.
The super-pangenome is the approach of developing a pangenome of the pangenomes of different species for a given genus.
Currently available methods and tools to develop pangenomes are mainly restricted to bacterial (prokaryotic) genomes. It is important now to develop novel, effective, and user-friendly tools for the development of super-pangenomes for crop (eukaryotic) genomes.

## Genome to Pangenome: A Paradigm Shift

The increasing population, unpredictable global climatic changes, and the emergence of resistant pathogens are serious threats to food security. The current trend of climate changes is expected to have adverse environmental effects, such as frequent occurrences of drought and other extreme weather events, which will largely hinder crop production and the availability of food in the majority of developing countries [1,2]. The world population is expected to reach 9.8 billion by 2050 and it is estimated that food production needs to be increased by 70% until 2050 to feed the increasing population (https://population.un.org/wpp/) [3]. One of the ways to ensure food security is to develop crop varieties that are sustainable and have the ability to adapt to changing environments. Currently, there is a huge gap between actual crop productivity and its yield potential, which could be tapped into by developing stress-resilient varieties to increase the productivity and quality of crops and achieve global food security targets by 2050.

The majority of efforts in recent decades to increase crop productivity focused on conventional breeding approaches like phenotyping-based selection. However, the past 10 years have witnessed a rapid evolution of marker technology and marker-based breeding approaches. Researchers have used advanced technologies such as **genomics-assisted breeding (GAB)** (see Glossary) and genetic engineering to develop modern crop varieties [4]. However, to deploy GAB and/or genetic engineering to develop superior varieties, the identification of markers/loci/genes associated with traits of interest is a prerequisite [4]. Sequencing and genotyping have become more readily available and affordable with the significant advances in **next-generation sequencing (NGS) technologies**, thus boosting the use of genomics for crop improvement [5,6]. Numerous sequencing efforts have been undertaken in plants and, as a result, reference genome sequences have become available for several crops, which serve as a base for crop improvement efforts [7, 8, 9, 10, 11, 12]. In addition to draft/reference genomes, several resequencing efforts have been initiated to capture the genetic diversity available in many cultivated and wild gene pools [13, 14, 15, 16]. Resequencing of cultivated lines helps in understanding the genetic diversity present in the species in terms of SNPs and small insertions/deletions (InDels) that can be used as markers to develop robust varieties through GAB. However, it has been noted that the studies that were largely focused on SNPs/InDels are not sufficient to represent the complete genetic repertoire of a species, as these variations alone do not contribute to the genetic diversity [17,18]. Recent studies identified another source of variations called **structural variations (SVs)**, which are known to play an important role in plant genetics and include **presence/absence variations (PAVs)**, **copy number variations (CNVs)**, and other, **miscellaneous variations** in the form of inversions, transversions, and inter/intrachromosomal translocations [19, 20, 21, 22]. Several resequencing studies in crop plants have identified SVs [16,23, 24, 25]; however, few comprehensive efforts have been made towards harnessing the potential of SVs in crop improvement.

Crop evolution and domestication have drastically reduced genetic diversity, resulting in the loss of several loci controlling important traits [26, 27, 28]. Intensive breeding processes that involve the selection of desirable traits such as resistance to a particular disease or tolerance to abiotic stress to enhance crop productivity have further aggravated the situation, leading to the loss of several other disease-resistance traits in the cultivated gene pool that were present in **crop wild relatives (CWRs)** [29, 30, 31, 32]. Because of this, the crops became more susceptible to various stresses like diseases and pests and to the effects of climate change. To overcome these vulnerabilities, there is a need to move towards the wild relatives of crops, which are known to possess genes for several important traits like tolerance to various stresses that have been lost during domestication or breeding processes [28,33, 34, 35, 36, 37]. The genetic material of CWRs can serve as a source of resistance/tolerance to the different stresses and can be introgressed in cultivated lines to expand their genetic base. Although crop improvement by utilizing wild species is a demanding task, due to the possibility of linkage drag, it is still achievable owing to recent technological advances. For instance, the CRISPR–Cas9 **genome editing** strategy has been deployed to integrate agronomically desirable traits of cultivated tomato (*Solanum lycopersicum*) with useful properties of wild relatives to develop better varieties [38].

Recent resequencing efforts exploring the huge genetic diversity present across diverse accessions were limited by the use of a single reference genome (for a given species), because mapping of the reads on the reference genome tends to miss highly polymorphic regions and regions that are not present in the reference genome [14,24]. A more robust and comprehensive approach is desired to capture all variations in a species. One such approach, which seems to be promising in representing the complete genetic repertoire of a species, is pangenomics. The concept of the **pangenome** was introduced for the genome analysis of multiple pathogenic isolates of *Streptococcus agalactiae* [39]. A pangenome broadly comprises two parts: the **core genome** and the **dispensable genome**. There are two types of pangenome: **open** and **closed**. The studies in plants have revealed that generally the core genome is bigger in size and has the maximum share of the genes [40, 41, 42]. It is believed that the dispensable genome may contain genes responsible for adaptation and survival in different environments. The comparison of the core genome of wild species and the dispensable genome of cultivated species uncovers the effect of domestication [40]. Pangenome analysis also helps in the identification of genes that are missing in reference genomes. First introduced in prokaryotes, pangenome studies are now gaining popularity in plant species as well [40, 41, 42, 43]. The studies in prokaryotes and eukaryotes have clearly demonstrated the need for a pangenome, as a single reference genome is not adequate to represent the complete genomic repertoire of a species [44,45].

In this opinion article, we discuss the need for pangenomes in crops, the recent developments in various plant species, and the critical role of CWRs in pangenome establishment. We also describe the different approaches available for pangenome analysis and factors critical in generating a pangenome.

## SVs Drive the Dispensable Genome

Genetic variations deployed as molecular markers have been of great interest in plant breeding. A wide range of molecular markers, including restriction fragment length polymorphism, random amplified polymorphic DNA, amplified fragment length polymorphism, simple sequence repeats (SSRs), and intergenic SSRs, have been developed and used over the past few decades. In previous decades, microsatellites or SSRs were widely used markers because of their codominance, multiallelic, highly polymorphic nature, and easy genotyping [46, 47, 48]. However, with advances in sequencing technologies, polymorphisms at the single-nucleotide level could also be identified, leading to the development of more robust SNP markers. In the current scenario, SNPs are the preferred choice of markers because of low cost and the amenability of automation. Further, advances in genomics and the availability of a large number of sequenced genomes increased our interest in resolving the genetic differences in terms of SVs.

SVs are highly abundant in human genomes and their association with diseases has also been established [49,50]. The recent studies pertaining to SVs in plants have demonstrated their importance in plant genetics as well [18,51]. Linking of genes with phenotypic traits has been immensely useful for GAB in crops [19,51,52]. Several studies have clearly demonstrated the role of SVs in deciphering the phenotype and orchestrating the mechanism of defense response in many plant species (Table S1 in the supplemental information online). Being subject to selective pressure, SVs form an integral part of the evolutionary process of a given species. The genes present in these SVs may be present across just one of the accessions and might be responsible for resistance to stress and pathogens (Table S1). For example, sequencing of the flow-sorted 3B chromosome from a hexaploid wheat (*Triticum aestivum*) genotype and its comparative analysis with the Chinese spring genome identified 159.3-Mbp SVs that might be associated with adaptation in wheat [53]. To capture the genetic diversity within a species, which is mainly contributed by SVs, its pangenome needs to be developed.

## Pangenome Development: Approaches and Critical Factors

With the availability of genome sequence and resequencing data, pangenomics is gaining popularity among researchers as an approach to tap the complete diversity present in a species. The various approaches used for the construction of a pangenome are reviewed in Box 1. The development of the pangenome depends on important factors such as the selection of the accessions, the approach used to develop the pangenome assembly, the quality of the genome assembly, and accurate detection of SVs. To capture the maximum diversity for a species, accessions with diverse morphological, phenotypic, and geographical origins should be selected [40,43]. A limited number of diverse individuals can give realistic estimates of the pangenome compared with several closely related accessions that compromise on diversity. It is advised to include all the accessions with desired/positive phenotype/agronomic traits to develop pangenomes which can be used in breeding applications.

Box 1 **Approaches Available for the Pangenome**

Three different approaches have been used so far for pangenome development [79].

***De Novo* Assembly**

This approach includes high-depth sequencing of all of the targeted accessions followed by the generation of individual *de novo* assemblies for each accession. The individual assemblies generated are then compared for the identification of conserved and variable regions. This method aims to generate the individual genome assemblies without a reference genome. The assemblers, such as SOAPdenovo, ALLPATHS-G, and ABySS, have been used for the development of *de novo* assemblies for pangenome construction in plants [40,43,80].

**Reference-Based Assembly and Iterative Mapping**

In this approach, the sequencing reads are first mapped to the existing reference genome and then unmapped reads are assembled using *de novo* assemblers after removal of bacterial and other contaminants. The assembled contigs/scaffolds are then anchored to the existing pseudomolecules using the paired-end reads information. The remaining unanchored contigs/scaffolds and updated pseudomolecules collectively form the pangenome for the given species. This approach has been used to map resequencing data from accessions of various species [41,81,82].

**Graph and *k*-mer**

Many assemblers use graph-based algorithms such as de Bruijn and string graphs to assemble the reads to represent a genome. A genome may be represented as a graph to depict the regions where chromosomes differ. Similarly, a colored graph could be used to represent multiple genomes encapsulating all variations existing between these genomes and confiscating a set of all nonredundant contents of the representing genomes. A pangenome may also be represented as a set of *k*-mer sequences. A set of such *k*-mer sequences eventually results in a de Bruijn graph. The merit of using the *k*-mer approach to designate a pangenome is supported by the fact that the *k*-mer approach is robust, rapid, and straightforward. Tools like SplitMEM use suffix trees and the de Bruijn graph approach for pangenome analysis [83]. This approach has been used extensively for prokaryotic pangenomes, but for complex eukaryotic genomes its use is limited. The major bottleneck with de Bruijn graphs is that the large sequence will lead to a very high number of vertices, which in turn will lead to a graph whose size will be very large. These graphs will be highly computationally intensive [84].

Further, the use of the correct approach according to the data available for the study will highly affect the construction of a confident pangenome. The *de novo* assembly approach seems to have an additional advantage over the reference-based approach as it reduces the potential bias arising due to compelling differences in genome size and structure. It also minimizes the possibility of misalignment and takes care of the critical sequences that cannot be aligned with confidence to the reference genome. However, *de novo* assembly is computationally intensive and demands ample infrastructure to generate multiple *de novo* genome assemblies. Additionally, the high quality of genome assembly, the annotation of gene models and the accurate detection of SVs significantly affect the quality of the pangenome. Accurate assemblies with greater coverage can be developed using recent technologies like the NRGene assembly, Hi-C, 10x Genomics, PacBio, Nanopore, etc. [8,11,12,54, 55, 56].

SV detection can be erroneous owing to sequencing artifacts and the presence of chimeric reads. The presence of repetitive regions in the genome further adds to the complexity in SV detection. A number of tools based on split-read, read-pair, read-count, and *de novo* assembly approaches are available for SV detection [57, 58, 59, 60, 61]. Based on all of these factors, a number of tools have been designed for pangenome analysis (Table S2 in the supplemental information online; reviewed in Box 2).

Box 2 **Key Tools for Pangenome Analysis**

Since the concept of the pangenome was first introduced in bacteria, the majority of the available pangenome tools, such as Panseq, PGAT, BPGA, etc., are for prokaryotic species [85, 86, 87]. These tools can handle genomes of smaller size and lower complexity. With an ever-increasing number of samples being sequenced regularly, there is an immediate need to develop a framework to store the genome sequences and update the pangenome for a species with every new variety added in the sequencing list. The pangenome information must not be restricted to only the gene level but should be extended to the whole-genome level. There are few tools available for eukaryotic pangenome analysis. EUPAN is a eukaryotic pangenome analysis toolkit, which facilitates the pangenome analysis of high-throughput data generated for eukaryotes [88]. The tool enables analysis of the data at low sequencing depth to construct pangenome. EUPAN was used to analyze 453 rice genomes, which resulted in the development of the pangenome and eventually the presence of PAVs across these genomes. EUPAN has been developed using Perl, R, and C++ and is supported for Linux platforms. Another tool, GET_HOMOLOGUES-EST, was developed to analyze the large-size plant genomes [89]. It handles the redundant and fragmented transcripts from RNA-seq data and incomplete gene models predicted for *de novo* genome assemblies. Similarly, the graph-, string-, multiple sequence alignment-, and *k*-mer-based frameworks may be deployed to store, analyze, and query the pangenome for large plant genomes. Currently, few tools are available that have the capacity to call variants using pangenome data structure. One such tool is PanVC (https://gitlab.com/dvalenzu/PanVC), which uses the pangenomic reference as a multiple sequence alignment, indexes the pangenome, finds the heaviest path, and calls variants. A similar tool, CHIC aligner (https://gitlab.com/dvalenzu/CHIC), is an aligner focusing on repetitive references. This tool is designed to map the individual reads to the pangenome (multiple reference genomes). GenomeMapper is another such tool, which supports simultaneous alignment of short reads against multiple reference genomes [90]. The various tools available for pangenome analysis are summarized in Table S2 in the supplemental information online.

## Walking on the Wild Side by Exploiting CWRs

CWRs have high genetic diversity and a very high potential of surviving in natural environments as compared to their cultivated counterparts [2,28,42]. The domestication and breeding processes have resulted in crops that feed the population today at the cost of reducing the genetic variation in these crops. There are several cases of selective sweep observed due to positive selection of a genomic locus controlling a desired trait, which resulted in reduction of diversity [62, 63, 64, 65].

Approximately 50 000–60 000 species of CWRs are currently known, of which nearly 10 000 may be considered of high potential for food security [33]. CWRs are known to be capable of attenuating the impact of changing climates as their genetic composition provides greater tolerance to drought, salt, and other abiotic stresses [66, 67, 68]. A number of studies have reported the use of CWRs to improve crop performance, thus establishing their robustness as potential targets for food security (Table S3 in the supplemental information online).

Sequencing approaches have enabled better understanding of genetic architecture of CWRs, thus facilitating their use in crop improvement. The completion of genome sequencing of major crop species has uncovered the need for a wider gene pool, which can be achieved by targeting CWRs. In the past decade, several studies deploying *de novo* assembly and resequencing approaches utilizing CWRs have been reported. Resequencing of 14 cultivated and 17 wild accessions of soybean (*Glycine max*) confirmed greater allelic diversity present in wild than in cultivated and identified high linkage disequilibrium in soybean [13]. In another study, resequencing of cultivated and wild accessions highlighted the alterations in the genetic constitution of soybean during domestication. This study reported 230 selective sweeps and 162 CNVs, some of which were linked to important agronomic traits like oil content and biotic resistance [24]. Similarly, in rice (*Oryza sativa*), 40 cultivated and ten wild accessions were resequenced, resulting in the identification of genes showing different genetic diversity levels among wild and cultivated accessions. These genes were related to domestication, disease resistance, and flowering [63]. Furthermore, a rice variation map constructed from 446 accessions of the wild rice species *Oryza rufipogon* was reported, which underlined loci linked to domestication related genes including hull color, seed shattering, and grain width [69]. In another study, 75 maize (*Zea mays*) lines, including wild, landrace, and improved, were resequenced to assess the evolution of modern maize. The study highlighted a number of genes linked to selection and provided evidence for introgression from wild relatives [70]. In pepper (*Piper nigrum*), 2.6% of the genome harbored strong selective sweep signals related to disease resistance, fruit ripening, seed dormancy, and transcription factors like ethylene responsive factor and basic helix-loop-helix [71].

**Figure 1:**
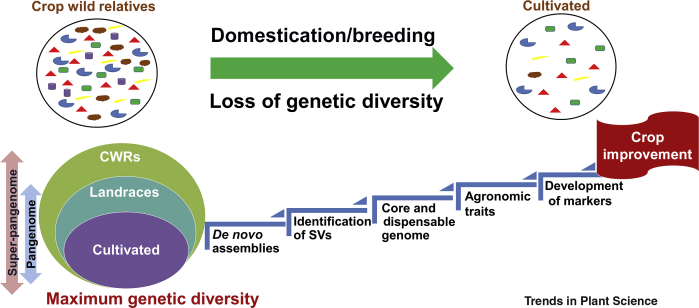
The Concept of the Pangenome and Super-Pangenome and Their Use for Crop Improvement. During the process of domestication and breeding, a number of alleles were lost. Therefore, it is important to have a catalog of all possible alleles in a crop species pangenome or genus-level super-pangenome. The pangenome and super-pangenome can be used for the development of markers using genome-wide association studies. These molecular markers can be used for crop improvement using genomics-assisted breeding approaches. Abbreviations: CWRs, crop wild relatives; SVs, structural variations.

The dynamic resources available from these findings can be utilized for crop improvement in respective species. Further, sequencing of the cultivated and the wild followed by the identification of common and specific regions through pangenome analyses can be a robust step towards better understanding of the wilds (Figure 1).

## Current Status of the Pangenome in Crop Plants

Recently, pangenomes have been developed for several crops such as rice, soybean, wheat, sesame (*Sesamum indicum*), and tomato. These studies have highlighted that SVs are a major source of variations and the use of a pangenome eliminates single-sample bias and has the capacity to present a nearly full view of the diversity present in a species [40, 41, 42,72,73].

In one of the early initiatives, a pangenome from seven phylogenetically and geographically different accessions of *Glycine soja*, a wild relative of *G. max*, was reported [40]. In the study, seven different accessions were assembled using a *de novo* assembly approach and ∼80% of the pangenome was conserved across all of the accessions. The remaining 20% of genomic content exhibited considerable variation in the form of SVs, indicating its involvement in plant adaptation to diverse surroundings and conditions. InDels with frameshift mutations were found to affect genes such as *Spiral2*-homolog, which is believed to provide twining growth habit in *G. soja*, in contrast to erect growth in *G. max*. Intergenomic comparisons further identified 1978 genes affected by CNVs. PAV analysis suggested the presence of 2.3–3.9-Mbp *G. soja*-specific PAVs in *G. soja* genomes. Overall, the SVs identified were found to have strong associations with important agronomic phenotypes such as flowering time, seed composition, organ size, and resistance [40]. Similarly, numerous efforts have been made to develop pangenomes from diverse rice accessions by different research groups, which are reviewed in Box 3.

Box 3 **Significance of the Pangenome Highlighted Through Various Studies in Rice as an Example**

Considering the inefficiency of a single reference genome, *de novo* assemblies of three divergent rice accessions were generated and compared with the Nipponbare reference genome. As a result, many genome specific regions containing functional genes were identified in this study, which would have been missed by a standard reference alignment strategy. In addition, the study unraveled many genomic regions linked with agriculturally important traits, such as the *Sub1* submergence-tolerance locus, the *LRK* gene cluster known to be associated with improved yield, and the *Pup1* cluster associated with phosphorus deficiency. The study emphasized the importance of *de novo* assembly approaches for pangenome development [43]. In another study, a *de novo* assembly approach was used to construct a dispensable genome from 1483 cultivated rice accessions [81], which led to the identification of genes related to important agronomic traits. These genes were found to be missing in the Nipponbare reference genome. Further, genome-wide association studies were conducted using SNPs between the dispensable sequences of different rice accessions for grain width and metabolic traits. About 23.5% of the metabolic traits showed significant association signals with polymorphisms from dispensable sequences than with SNPs from the reference genome and 41.6% of trait-associated SNPs had concordant genomic locations with associated dispensable sequences. The 3000 Rice Genomes Project was used to develop an interactive web-based pangenome browser, ‘The Rice Pan Genome Browser’ [82]. In the study, reads from 3010 accessions were mapped on the IRGSP-1.0 genome, which identified a total of 23 914 core genes, 4986 candidate core genes, and 22 095 distributed genes. Of the distributed genes, 853 genes were subspecies or varietal group specific, including 587, 147, 67, and 52 genes specific for *Indica* and *Japonica* subspecies, *Aus* and *Aro* groups, respectively. Notably, ∼12 000 novel genes absent in the reference genome were reported in the study. Recently, a pangenome for the *Oryza sativa* and *Oryza rufipogon* species was reported. In this study, 66 diverse accessions were deep sequenced and their individual *de novo* assemblies were developed. The pangenome of these assemblies was able to capture six more domestication sweeps that were missed by previous studies. The PAV profiling resulted in 10 872 genes in the 67 rice accessions that were partially absent in the Nipponbare reference [42].

The pangenome for *Brassica oleracea* was constructed by an iterative mapping and assembly approach using eight cultivated and one wild accessions [41]. The size of the pangenome was 587 Mbp and it contained 61 379 gene models, of which 81.3% were part of the core gene set. Modelling of the pangenome indicated that the *Brassica* pangenome is a closed pangenome, similar to soybean and maize [40,74]. In addition, functional analysis revealed variations in agronomically important genes such as auxin-related genes, flowering-related genes, disease resistance, and glucosinolate metabolism [41].

Further, the wheat pangenome was reported using an approach similar to that in *Brassica* [72]. The large size and the high number of repeat elements contribute to the complexity of the wheat genome [10]. The assembly of such genomes using *de novo* assembly approaches remains a challenging task even with advances in technology [75]. Therefore, in the study, an improved version of the Chinese spring genome assembly with increased size and decreased duplicated regions was developed. Further, 18 wheat cultivars were mapped to this assembly resulting in 221 991 newly assembled scaffolds with a total length of 350 Mbp and 21 653 predicted genes. The PAV analysis revealed that the pangenome of modern wheat cultivars has 140 500 ± 102 genes and an average of 49 unique genes per cultivar. Gene Ontology (GO) enrichment analysis of the dispensable genome suggested enrichment of genes related to stress and defense responses [72].

In the case of poplar (*Populus*), a comprehensive study of SVs was conducted using three intercrossable species: *Populus nigra*, *Populus deltoides*, and *Populus trichocarpa*. Using *P. trichocarpa* as the reference genome, a total of 7889 insertions and 10 586 deletions were identified. The study indicated that SVs result in the genetic variability of poplar and the InDels were found to affect roughly 20% of the poplar genome. Based on the SV analyses, the pangenome size for poplar was estimated to be ∼497 Mbp, with 80.7% constituting the core genome, similar to other pangenome studies [40,72]. The study suggested that increasing the number of individuals will result in expansion of the dispensable genome for poplar as it is highly affected by private variants [76].

A sesame pangenome of 554.05 Mbp with core and dispensable genomes of 258.79 Mbp and 295.26 Mbp, respectively, was reported. The sesame pangenome highlights an instance where the sizes of the core and dispensable genomes are comparable. The pangenome was constructed from five sesame varieties, which included two landraces and three modern cultivars. The pangenome comprised 26 472 orthologous gene clusters of which 58.21% were core. The comparative evolutionary analysis presented in the study suggested the putative involvement of genes related to plant–pathogen interaction and lipid metabolism in promoting high accumulation of oil and fatty acid in sesame seeds and hence improved environmental adaption [73].

A pangenome for sunflower was constructed using 287 cultivated lines. The reads from these accessions were mapped onto the sunflower reference genome and the unmapped reads were *de novo* assembled to develop a pangenome for cultivated sunflower. The pangenome comprised 62 205 genes of which 32 917 represented core genes. From the set of dispensable genes, 2464 were found in less than 5% of the accessions. Along with cultivated, the study also sequenced 189 wild accessions, which were compared with the pangenome, and it was observed that 10% of the cultivated pangenome is derived through introgression from wild species. Further, functional annotation of the introgressed genes revealed that these genes were mainly related to biotic resistance, supporting the finding that the wild relatives of sunflower contribute to its disease resistance [77].

Recently, a tomato pangenome was developed from 725 geographically and phylogenetically diverse accessions. The ‘map-to-pan’ strategy resulted in the identification of 351 Mbp of sequences (comprising 4873 novel genes) missing in the reference genome. The modelling of the tomato pangenome indicated it to be a closed pangenome with finite numbers of core and dispensable genes. The pangenome analysis resulted in the identification of a 4-bp substitution in the regulatory region of the *TomLoxC* gene modifying the tomato fruit flavor. Overall, the study suggested that human selection altered fruit quality and other phenotypes by affecting the regulatory sequences [78].

## Super-Pangenome: A Way Forward

To date, pangenome studies have largely focused on the use of different cultivated accessions of a crop. Such pangenomes do not represent a sufficiently diverse germplasm, as these cultivated accessions belong to one species and hence these pangenomes could be considered subpangenomes of the genus. For a comprehensive pangenome, it is important that we move towards utilizing the genus-level pangenomes. As different species in a genus are available for a given crop, useful genes can be transferred from one species to another either simply by a crossing mechanism, especially with species from the secondary gene pool, or by wide hybridization or modern chromosome/genome engineering approaches for species belonging to other/distantly related gene pools.

**Figure 2:**
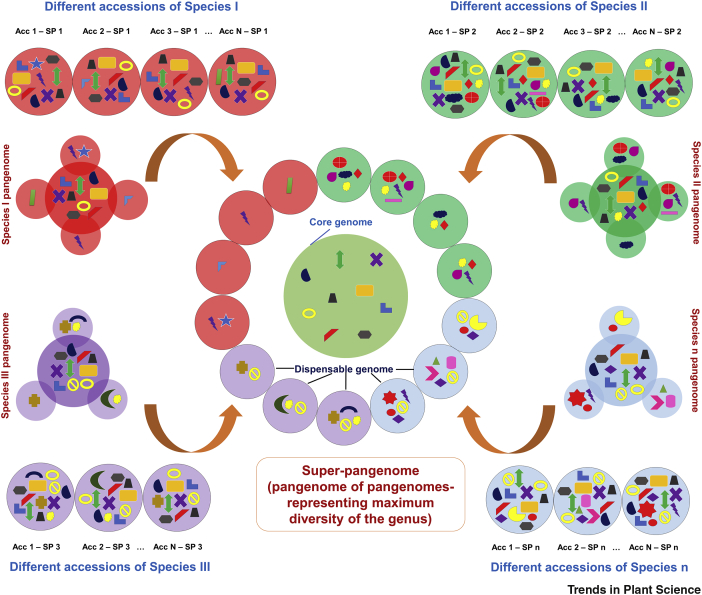
Key Figure. Approaches for the Construction of a Super-Pangenome The figure presents a schema for the construction of a super-pangenome using diverse accessions of all of the species of a given genus. A minimum of ten different accessions for each species can be used to construct a species-level pangenome, and combining these species pangenomes would result in a super-pangenome, which would ultimately have the capacity to represent the complete genetic repertoire of the genus, thus providing a vast resource for the acceleration of crop improvement. Abbreviations: Acc, accession; SP, species of a given genus.

Considering the advances in NGS technologies with reducing cost, we propose a more comprehensive approach where we strive for a super-pangenome (Figure 2, Key Figure). This approach starts with the identification and selection of the most diverse accessions from a particular species (say, Species I), followed by *de novo* genome assembly of one of the accessions and then mapping of resequencing data from the remaining accessions onto this assembly to construct a species-level pangenome (Species I pangenome). For instance, from Species I, select the ten most diverse accessions, then assemble one of these accessions and map the sequencing data from the other nine accessions on this assembly to develop a pangenome, which will represent the genetic makeup of Species I. Similarly, a pangenome for another species (e.g., Species II) will be constructed. In this way, different species-level pangenomes will be generated for the genus of the given crop species. The pangenome for a genus would be developed by combining these species-level pangenomes. The pangenome thus constructed will be called a super-pangenome and will have the potential to represent the complete genetic repertoire of the genus. We propose to generate the super-pangenome by developing at least one *de novo* assembly from each species, as it reduces the bias of mapping the sequencing data from accessions of other diverse species. Also, we suggest adding at least ten diverse accessions from each species to develop the species-level pangenomes for all of the species. The study of such pangenomes will provide better insights into genes present/absent across the different species and help to decipher genetic material specific to the species/gene pool/lineage. A more comprehensive coverage of genes in the dispensable genome enhances the process to pinpoint genes associated with important agronomic traits such as disease resistance, seed composition, maturity, flowering time, and organ size, thus enabling its use in accelerating breeding programs. Considering the fact that the super-pangenome will have the capacity to represent a complete genus, it can be speculated that the size of the core genome, which is usually considered to be the dominant part of the pangenome, may not be the major fraction. The super-pangenome will also serve as an excellent resource for evolutionary studies as it will enable accurate detection of the divergence time between the species and provide a true estimate of the different evolutionary events shaping the present genomic architecture of different species. The super-pangenome would discern novel haplotypes of potential use for future crop improvement and conservation efforts.

## Concluding Remarks

In summary, pangenome development is imperative for in-depth dissection of dispensable as well as species-specific genes. It could help to identify genes involved in adaptation and help in the formulation of strategies for the introduction or cultivation of environmentally stable varieties. The variations identified through pangenome analysis can be used as markers for **marker-assisted selection**, by which desirable traits present in CWRs can be incorporated into domesticated cultivars. A super-pangenome aims to represent the complete genetic architecture of a genus by combining the different pangenomes from all of the species of the given genus. When the diverse accessions from different species are superimposed, the complete genetic repertoire would be achieved. The implementation of the super-pangenome concept will definitely boost GAB and will enhance the crop improvement process (see Outstanding Questions).

Outstanding Questions How can we implement pangenomics-assisted breeding for crop improvement?

How can we translate information from the super-pangenome into the development of improved crop varieties?

Can super-pangenome information be linked to epigenomics to address more complex biological questions, such as gene regulation?

Can we develop highly interactive open-source visualization tools with the capacity to represent the super-pangenome?

Can we build an efficient framework with the ability to store and retrieve large amounts of data for pangenome analysis?

## Glossary

**Closed pangenome**: a type of pangenome in which the size does not increase after a certain number of individuals are added.
**Copy number variations (CNVs)**: the genomic regions with differences in copy number among individuals.
**Core genome**: the part of the pangenome that is shared among all individuals of a species.
**Crop wild relatives (CWRs)**: includes both the crop ancestor as well as related species which have not been domesticated and may contain the alleles for the stress resilience and agronomic traits which are not present in the cultivated genepool.
**Dispensable/variable genome**: the part of the pangenome that is present in some individuals but not all.
**Genome editing**: technologies that enable modifications in the DNA sequence including adding, removing, or altering bases in an organism.
**Genomics-assisted breeding (GAB)**: integration and use of genomics tools/information in breeding programs to develop elite lines with enhanced yield, biotic/abiotic stress tolerance, and better nutrition.
**Marker-assisted selection**: method that deploys molecular markers for the selection of desirable individuals in a breeding program; especially used for traits that cannot be easily selected using conventional approaches.
**Miscellaneous variations**: genomic variations in the form of inversions, transversions, and inter/intrachromosomal translocations.
**Next-generation sequencing (NGS) technologies**: various modern high-throughput technologies that allow rapid and cost-effective sequencing of both DNA and RNA.
**Open pangenome**: a type of pangenome in which the size of the pangenome tends to increase with the addition of each individual.
**Pangenome**: the total genome architecture of a species developed by the sequencing and analysis of multiple accessions of a species.
**Presence/absence variations (PAVs)**: the genomic regions that include the sequences completely missing in one of the individuals.
**Structural variations (SVs)**: genomic variations in DNA segments of more than 1 kbp.
