# Supplementary material for: Super-Pangenome by Integrating the Wild Side of a Species for Accelerated Crop Improvement
Source: Trends Plant Sci. 2020 Feb;25(2):148–58. doi: 10.1016/j.tplants.2019.10.012 (PMC6988109; doi:10.1016/j.tplants.2019.10.012)
Supplement: Supplementary Material [file mmc1.docx]

*Supplemental Information*

**Super-Pangenome by Integrating the Wild Side of a Species for Accelerated Crop Improvement**

Aamir W. Khan^1,2^, Vanika Garg^1^, Manish Roorkiwal^1^, Agnieszka A. Golicz^3^, David Edwards^2^, Rajeev K. Varshney^1,^**^*^**

^1^Center of Excellence in Genomics & Systems Biology, International Crops Research Institute for the Semi-Arid Tropics (ICRISAT), Hyderabad, India

^2^School of Biological Sciences, The University of Western Australia (UWA), Crawley, Western Australia, Australia

^3^School of Agriculture and Food Sciences, The University of Queensland, Brisbane, Queensland, Australia

^*^Correspondence: r.k.varshney@cgiar.org (R.K. Varshney)

**Table S1. Key studies highlighting association of structural variations with important traits in plants**

| **Plant**  **Species** | **Structural variations (SVs)** | **Number of accessions** | **Target traits** | **Method** | **Tools** | **Refs.** |
| --- | --- | --- | --- | --- | --- | --- |
| **Arabidopsis (*Arabidopsis thaliana*)** | 1,059 CNVs | 80 inbred lines | Adaptation to diverse environments | Resequencing and *de novo* assembly | BWA | [45] |
| **Grape**  **(*Vitis vinifera*)** | 310,855 insertions, 312,148 deletions, 5,871 complex SVs | 1 genotype | Seedlessness and embryo development | *de novo* assembly and reads mapping methods | BWA, Dindel, Nucmer | [S1] |
|  | ~700 CNVs | 4 table grape cultivars | Primary metabolism and stress responses like chitinases | Sequencing, array comparative genomic hybridization, FISH, qPCR | BWA, GATK | [S2] |
| **Maize**  **(*Zea mays*)** | SV on chr 6 | 8 inbred lines | Grain yield | QTL mapping via MAGIC population | GenomeStudio | [S3] |
|  | Tandem triplication of *MATE1* gene | - | Aluminium tolerance | qPCR, FISH | - | [S4] |
|  | 147-kb deletion of ZmWAK | - | Resistance to head smut | Fine mapping and transgenic complementation | - | [S5] |
| **Medicago (*Medicago truncatula*)** | 500,000–1,500,000 short InDels 49,000–169,000 CNVs 2,700–12,700 translocations | 15 accessions | NBS-LRR and RLK genes related to resistance | *de novo* assembly and pan genome approach | OrthoMCL, Bcftools | [S6] |
| **Potato (*Solanum tuberosum*)** | CNV calls per individual varying from 2,978 to 10,532 | 12 monoploid/doubled monoploid clones | Adaptation and tolerance to abiotic stress and herbicides | Genome resequencing, transcriptomics, FISH | CNVnator | [26] |
| **Rice**  **(*Oryza sativa*)** | 7,393 large insertion/deletion (between Kasalath and Nipponbare), 3,780 large InDels (between Kasalath and 93-11) | 1 (*aus* rice cultivar Kasalath) | QTL for phosphorus-deficiency tolerance | *de novo* assembly of Kasalath sequences | MUMmer, BWA | [S7] |
|  | CNV on Chromosome 7 | - | Grain size diversity | - | - | [22] |
|  | 131 large inversions, 4,509 PAVs specific in ZS97RS1 and 4,566 in MH63RS1 | 2 lines | Plant height, number of grains and stress response | BAC sequencing using PacBio | MUMmer | [S8] |
| **Sorghum (*Sorghum bicolor*)** | 51 large-size (>30 kb) PAVs covering 2.92 Mb of the sorghum genome | 4 inbred lines | Cell death and stress response | - | - | [S9] |
|  | 5,511 PAVs affecting 3,238 genes | 4 inbred lines including 2 grain sorghum | Genes for nucleotide binding and protein modification by ubiquitination | Whole genome resequencing | SOAPaligner, SOAPcoverage | [S10] |
| **Soybean (*Glycine max*)** | 10,928 medium-size deletions | 10 cultivated, 6 wild | Domestication and seed, flower development related | Whole genome resequencing | SOAPdenovo, GATK | [S11] |
|  | - | 41 accessions | Adaptive traits, resistance (R) genes including leucine-rich repeat, nucleotide binding, and Toll-interleukin receptor protein domains | Array hybridization, whole genome resequencing | BWA | [S12] |
|  | 31 kb DNA segment exhibiting CNV associated with Rhg1 | 41 accessions | Soybean cyst nematode resistance | Whole genome sequencing, fiber-FISH | GATK, Bowtie2 | [S13] |
|  | 7,902 PAVs | 106 accessions | Acyl-lipid metabolism, protein content | Whole genome resequencing | SOAP2, SOAPsnp | [S14] |

**Table S2. Summary of key tools and pipelines used for pangenome analyses in different studies**

| **Tool Name** | **Platform** | **Language** | **Features** | **Refs.** |
| --- | --- | --- | --- | --- |
| PanGP | Linux, Windows, Web-based | C++ | Can handle huge number of samples using two different sampling algorithms and has a user friendly graphical user interface for pangenome profiling and visualization | [S15] |
| Micropan | Linux, Windows | R | A R package with multiple tools based on pan matrix data structure for a comprehensive pangenome analyses | [S16] |
| GET_HOMOLOGUES | Linux, Mac | Perl | An open source package for pangenome construction and comparative genomic analysis. A highly customized and automated pipeline especially designed people with non-bioinformatics background | [S17] |
| SplitMem | Linux | C++ | A robust graphical algorithm which utilizes de Bruijn graphs for visualization of different genomes | [84] |
| ITEP | Linux | Python | An integrated flexible toolkit designed for generation and curation of protein families, gene gain-loss analysis and metabolic network construction | [S18] |
| PANNOTATOR | Web-based | PHP, SQL, BioPerl | A fast, reliable and automated pipeline for the annotation of closely related and well-suited genomes | [S19] |
| PanOCT | Linux, Mac OS | Perl | A ortholog clustering tool designed for closely related species. Utilizes neighborhood information for separating recently diverged paralogs into orthologous clusters | [S20] |
| PanViz | Web-based | Javascript, R | An interactive visualization tool to compare the individual genomes to the pangenome. Requires no external program and allows visualization of groups of similar genes across genome | [S21] |
| Roary | Linux, Mac OS, Windows (Docker, VM available) | Python, Perl | A very high speed pipeline which accepts annotated assemblies in GFF3 format as input for pangenome construction. Not suitable for highly divergent genomes and metagenomics analysis | [S22] |
| Panseq | Linux, Web-based | Perl | One of the first tools for pangenomes analysis. Can determine core and accessory regions, identify SNPs followed by construction of phylogenetic trees | [87] |
| PGAP-X | Linux, Windows | C++/Qt | A genome-oriented software with four different modules for whole genome sequence alignments, orthologous genes clustering, pangenome profiling and genetic variant analysis | [S23] |
| Piggy | Linux | Perl, R | A rapid pangenome pipeline for detection of highly divergent intergenic regions | [S24] |
| seq-seq-pan | Linux | Python, Java | A framework that utilizes whole genome alignments as data structure and has the capacity to add or remove genomes from a aligned sequences | [S25] |

**Table S3. List of some crop wild relatives studies highlighting their association with important traits**

| **Crop** | **Crop wild relatives (CWRs)** | **Target trait** | **Approach/markers** | **Refs.** |
| --- | --- | --- | --- | --- |
| **Rice** | *Oryza rufipogon* | Grain quality | Backcross QTL analysis | [S26] |
|  | *Oryza officinalis* | Early morning flowering | Interspecific hybridization | [S27] |
|  | *Oryza rufipogon* | Nitrogen-deficiency tolerance | Chromosome segment substitution lines and SNPs | [S28] |
|  | *Oryza rufipogon* | Cold tolerance | QTL mapping and InDel markers | [S29] |
|  | *Oryza rufipogon* | Yield related | Introgression lines and SSRs | [S30] |
| **Barley** | *Hordeum bulbosum* | Resistance to scald | Recombinant Lines | [S31] |
|  | *ISR42-8, a wild barley accession* | Root dry weight, tiller number per plant, root length | Introgression lines and QTL mapping | [S32] |
| **Cassava** | *Manihot glaziovii* | Pest and disease resistance | Whole genome resequencing and QTL mapping | [S33] |
| **Cucumber** | *Cucumis sativus var. hardwickii* | Seedling traits | RILs, SSRs, QTL mapping | [S34] |
|  | *Gummy stem blight resistant wild accession, PI 183967* | Gummy stem blight | RILs, SSRs, QTL mapping | [S35] |
| **Tomato** | *Solanum habrochaites* | Resistance to late blight disease | Near-Isogenic Lines | [S36] |
|  | *Solanum pimpinellifolium* | Fruit quality | GBS and QTL mapping | [S37] |
| **Soybean** | *Glycine soja 85-32* | Aphid resistance | QTL mapping | [S38] |
|  | *Glycine soja* | Salt tolerance | Whole genome de novo sequencing, QTL mapping | [21] |
|  | *Glycine soja* | Soybean cyst nematode | GWAS | [S39] |
| **Wheat** | *Wild emmer wheat* | Grain yield, biomass, drought | Near-Isogenic Line | [S40] |
|  |  | Yield and root related | SNP genotyping, linkage mapping | [S41] |
| **Peanut** | *Arachis magna* | Rust resistance | QTL mapping | [S42] |
|  | *Arachis stenosperma* | Nematode resistance | Transcriptome analysis | [S43] |

**Supplemental References**

S1. Di Genova, A. et al. (2014) Whole genome comparison between table and wine grapes reveals a comprehensive catalog of structural variants. *BMC Plant Biol.* 14, 7

S2. Cardone, M.F. et al. (2016) Inter‐varietal structural variation in *grapevine* genomes. *Plant J*. 88, 648-661

S3. Dell’Acqua, M. et al. (2015) Genetic properties of the MAGIC maize population: a new platform for high definition QTL mapping in *Zea mays*. *Genome Biol*. 16, 167

S4. Maron, L.G. et al. (2013) Aluminum tolerance in maize is associated with higher *MATE1* gene copy number. *Proc. Natl. Acad. Sci. U.S.A.*110, 5241-5246

S5. Zuo, W. et al. (2015) A maize wall-associated kinase confers quantitative resistance to head smut. *Nat. Genet.* 47, 151-157

S6. Zhou, P. et al. (2017) Exploring structural variation and gene family architecture with *De Novo* assemblies of 15 *Medicago* genomes. *BMC Genomics* 18, 261

S7. Sakai, H. et al. (2014) Construction of pseudomolecule sequences of the *aus* rice cultivar Kasalath for comparative genomics of Asian cultivated rice. *DNA Res.* 21, 397-405

S8. Zhang, J. et al. (2016) Extensive sequence divergence between the reference genomes of two elite indica rice varieties Zhenshan 97 and Minghui 63. *Proc. Natl. Acad. Sci. U.S.A.* 113, E5163-E5171

S9. Zhang, L.M. (2014) Genome-wide patterns of large-size presence/absence variants in *sorghum*. *J. Integr. Plant Biol.* 56, 24-37

S10. Shen, X. et al. (2015) PAV markers in *Sorghum bicolor*: genome pattern, affected genes and pathways, and genetic linkage map construction. *Theor. Appl. Genet*. 128, 623-637

S11. Chung, W.H. et al. (2014) Population structure and domestication revealed by high-depth resequencing of Korean cultivated and wild soybean genomes. *DNA Res*. 21, 153-167

S12. Anderson, J.E. et al. (2014) A roadmap for functional structural variants in the soybean genome. *G3 (Bethesda, Md.)* 4, 1307-1318

S13. Cook, D.E. et al. (2014) Distinct copy number, coding sequence, and locus methylation patterns underlie *Rhg1*-mediated soybean resistance to soybean cyst nematode. *Plant Physiol*. 165, 630-647

S14. Valliyodan, B. et al. (2016) Landscape of genomic diversity and trait discovery in soybean. *Sci. Rep*. 6, 23598

S15. Zhao, Y. et al. (2014) PanGP: a tool for quickly analyzing bacterial pan-genome profile. *Bioinformatics* 30, 1297-1299

S16. Snipen, L. and Liland, K.H. (2015) micropan: An R-package for microbial pan-genomics. *BMC Bioinformatics* 16, 79

S17. Contreras-Moreira, B. and Vinuesa, P. (2013) GET_HOMOLOGUES, a versatile software package for scalable and robust microbial pangenome analysis. *Appl. Environ. Microbiol.* 79, 7696-7701

S18. Benedict, M.N. et al. (2014) ITEP: an integrated toolkit for exploration of microbial pan-genomes. *BMC Genomics* 15, 8

S19. Santos, A.R. et al. (2013) PANNOTATOR: an automated tool for annotation of pan-genomes. *Genet. Mol. Res.* 12, 2982-2989

S20. Fouts, D.E. et al. (2012) PanOCT: automated clustering of orthologs using conserved gene neighborhood for pan-genomic analysis of bacterial strains and closely related species. *Nucleic Acids Res.* 40, e172

S21. Pedersen, T.L. et al. (2017) PanViz: interactive visualization of the structure of functionally annotated pangenomes. *Bioinformatics* 33, 1081-1082

S22. Page, A.J. et al. (2015) Roary: rapid large-scale prokaryote pan genome analysis. *Bioinformatics* 31, 3691-3693

S23. Zhao, Y. et al. (2018) PGAP-X: extension on pan-genome analysis pipeline. *BMC Genomics* 19, 36

S24. Thorpe, H.A. et al. (2018) Piggy: a rapid, large-scale pan-genome analysis tool for intergenic regions in bacteria. *Gigascience* 7, 1-11

S25. Jandrasits, C. et al. (2018) seq-seq-pan: Building a computational pan-genome data structure on whole genome alignment. *BMC Genomics* 19, 47

S26. Septiningsih, E.M. and Trijatmiko, K.R. (2003) Identification of quantitative trait loci for grain quality in an advanced backcross population derived from the *Oryza sativa* variety IR64 and the wild relative *O. rufipogon*. *Theor. Appl. Genet*. 107, 1433-1441

S27. Ishimaru, T. et al. (2010) A genetic resource for early-morning flowering trait of wild rice *Oryza officinalis* to mitigate high temperature-induced spikelet sterility at anthesis. *Ann. Bot.* 106, 515-520

S28. Ogawa, S. et al. (2016) Identification of QTLs associated with agronomic performance under nitrogen-deficient conditions using chromosome segment substitution lines of a wild rice relative, *Oryza rufipogon*. *Acta Physiol. Plant* 38, 103

S29. Mao, D. et al. (2015) Multiple cold resistance loci confer the high cold tolerance adaptation of Dongxiang wild rice (*Oryza rufipogon*) to its high-latitude habitat. *Theor. Appl. Genet*. 128, 1359-1371

S30. Tian, F. et al. (2006) Construction of introgression lines carrying wild rice (*Oryza rufipogon* Griff.) segments in cultivated rice (*Oryza sativa* L.) background and characterization of introgressed segments associated with yield-related traits. *Theor. Appl. Genet*. 112, 570-580

S31. Pickering, R. et al. (2006) The transfer of a gene conferring resistance to scald (*Rhynchosporium secalis*) from *Hordeum bulbosum* into *H. vulgare* chromosome 4HS. *Plant Breed.* 125, 576-579

S32. Naz, A.A. et al. (2014) Wild barley introgression lines revealed novel QTL alleles for root and related shoot traits in the cultivated barley (*Hordeum vulgare* L.). *BMC Genetics* 15, 107

S33. Nzuki, I. et al. (2017) QTL Mapping for Pest and Disease Resistance in Cassava and Coincidence of Some QTL with Introgression Regions Derived from *Manihot glaziovii*. *Front. Plant Sci*. 8, 1168

S34. Wang, M. et al. (2016) QTL mapping of seedling traits in cucumber using recombinant inbred lines. *Plant Breed.* 135, 124-129

S35. Zhang, S. et al. (2017) Inheritance and QTL mapping of resistance to gummy stem blight in cucumber stem. *Mol. Breed*. 37, 49

S36. Haggard, J. E. and St.Clair D.A. (2015) Combining Ability for *Phytophthora infestans* Quantitative Resistance from Wild Tomato. *Crop Sci.* 55, 240-254

S37. Celik, I. et al. (2017) Genome-wide SNP discovery and QTL mapping for fruit quality traits in inbred backcross lines (IBLs) of *Solanum pimpinellifolium* using genotyping by sequencing. *BMC Genomics* 18, 1

S38. Zhang, S. et al. (2017) Mapping novel aphid resistance QTL from wild soybean, *Glycine soja* 85-32. *Theor. Appl. Genet*. 130, 1941-1952

S39. Zhang, H. et al. (2016) Genome-wide association study of resistance to soybean Cyst nematode (*Heterodera glycines*) HG type 2.5.7 in wild soybean (*Glycine soja*). *Front. Plant Sci*. 7, 1214

S40. Merchuk-Ovnat, L. et al. (2017) Ancestral QTL alleles from wild emmer wheat enhance root development under drought in modern wheat. *Front. Plant Sci*. 8, 703

S41. Lucas, S.J. et al. (2017) High-throughput SNP genotyping of modern and wild emmer wheat for yield and root morphology using a combined association and linkage analysis. *Funct. Integr. Genomic*. 17, 667-685

S42. Leal-Bertioli, S.C. et al. (2015) Identification of QTLs for rust resistance in the peanut wild species *Arachis magna* and the development of KASP markers for marker-assisted selection. *G3 (Bethesda, Md.)* 5, 1403-1413

S43. Guimaraes, P.M. et al. (2015) Root transcriptome analysis of wild peanut reveals candidate genes for nematode resistance. *PLoS One* 10, e014093
